# Increased rate of respiratory symptoms in children with Down syndrome: a 2-year web-based parent-reported prospective study

**DOI:** 10.1007/s00431-022-04634-1

**Published:** 2022-10-03

**Authors:** Noortje B. Eijsvoogel, Ruud H. J. Verstegen, Gijs Th. J. van Well, Roeland W. N. M. van Hout, Esther de Vries

**Affiliations:** 1 Tranzo Tilburg School of Social and Behavioral Sciences, Tilburg University, PO Box 90153 (RP219), 5000LE Tilburg, the Netherlands; 2 Department of Pediatrics, Maastricht University Medical Center, Maastricht, the Netherlands; 3 Division of Clinical Pharmacology and Toxicology, Department of Pediatrics, The Hospital for Sick Children, Toronto, ON Canada; 4 Division of Rheumatology, Department of Pediatrics, The Hospital for Sick Children, Toronto, ON Canada; 5 Department of Pediatrics, University of Toronto, Toronto, ON Canada; 6 Centre for Language Studies, Radboud University, Nijmegen, the Netherlands; 7 Jeroen Bosch Academy Research, Jeroen Bosch Hospital, ‘s-Hertogenbosch, the Netherlands

**Keywords:** Seasonal influence, Frequency of respiratory symptoms, Type of respiratory symptoms, Down syndrome, Parental questionnaire

## Abstract

To compare the incidence of respiratory symptoms and short-term consequences between children with Down syndrome and children from the general population, we conducted a prospective parent-reported observational study. Children with Down syndrome (≤ 18 years) were included between March 2012 and June 2014. Caregivers received a baseline questionnaire with follow-up 1–2 years after inclusion. Caregivers received a weekly questionnaire about respiratory symptoms, fever, antibiotic prescriptions, doctor’s visits, and consequences for school and work attendance. Children with Down syndrome were compared to a cohort of the general population (“Kind en Ziek” study) with similar weekly questionnaires. A total of 9,011 childweeks were reported for 116 participants with Down syndrome (75% response rate). The frequency of respiratory symptoms was higher in children with Down syndrome than in children from the general population (30% vs 15.2%). In addition, symptoms subsided later (around 8 vs 5 years of age). The seasonal influence was limited, both in children with Down syndrome and children from the general population. Consequences of respiratory disease were significant in children with Down syndrome compared to children from the general population, with a higher rate of doctor’s visits (21.3% vs 11.8%), antibiotic prescriptions (47.8% vs 26.3%), and absenteeism from school (55.5% vs 25.4%) and work (parents, 9.4% vs 8.1%).

*  Conclusion*: Children with Down syndrome have a higher frequency of respiratory symptoms and symptoms last until a later age, confirming the impression of professionals and caregivers. Individualized treatment plans might prevent unfavorable consequences of chronic recurrent respiratory disease in children with Down syndrome.

**Table:** 

**What is Known:** *• Children with Down syndrome have an altered immune system and are prone to a more severe course of respiratory tract infections*. *• The overall conception is that patients with Down syndrome suffer from respiratory tract infections more often*.	
**What is New:** *• Children with Down syndrome suffer from respiratory symptoms more frequently than children from the general population*. *• The respiratory symptoms in children with Down syndrome subside at a later age compared to children from the general population*.

**Supplementary Information:**

The online version contains supplementary material available at 10.1007/s00431-022-04634-1.

## Introduction

Down syndrome is the most common chromosomal abnormality among live-born infants (approximately 1 in 800 babies) [1]. Children with Down syndrome are known to have many concomitant health problems. Among these, recurrent respiratory infections contribute to increased morbidity and mortality in this population [2, 3]. Children with Down syndrome have a higher risk of a severe course of infections compared to children from the general population [3–6], resulting in more hospital as well as intensive care admissions [3, 6, 7]. Besides, milder respiratory and ear-nose-throat (ENT) infections recur frequently in children with Down syndrome [6, 8–10], which may cause hearing problems and aggravate obstructive sleep apnea syndrome [4, 8, 9].

It is a commonly reported observation by parents and health care professionals that the incidence of respiratory symptoms is increased in children with Down syndrome. However, their incidence has been poorly studied outside of the context of a severe disease course. One study showed that parents report more symptoms of recurrent wheeze, cough, and other respiratory symptoms in their children with Down syndrome compared to their siblings without Down syndrome [11]. Another study reported a higher incidence of respiratory symptoms in the two weeks preceding the parental interview [12]. However, prospective, longitudinal data are lacking.

Here, we describe the incidence and pattern of respiratory symptoms in children with Down syndrome as reported by parents or caregivers in a prospective nationwide web-based 2-year weekly survey and compare these data to a large population-based cohort of children from the general population [13]. The primary aim of this study is to obtain information on the frequency of respiratory symptoms in relation to age and season. Secondary aims include demonstrating the short-term medical and social consequences of respiratory symptoms, such as doctor’s visits, absence from school, and care leave of parents from work.

## Methods

### Study design

We conducted a prospective nationwide web-based parent-reported observational study (checklist STROBE guidelines in Supplemental Table 1); participants were included from March 2012 to June 2014. The methods were described in detail previously [14]. In short, parents or legal guardians were approached via specialty outpatient clinics for Down syndrome, social media, and the Dutch Down Syndrome Foundation. After online registration and informed consent, parents or legal guardians received a weekly invitation by email with a link to the online questionnaire, which was sent by an automated data management system (Research Manager, Cloud9 Software, the Netherlands). All questionnaires used in this study are included in Supplemental Table 2. The first questionnaire contained baseline questions regarding the composition of the household, the medical history of the child and family members, as well as the child’s daily activities. This questionnaire was repeated after one and two years. After the first baseline questionnaire was completed, parents received a weekly questionnaire regarding the presence or absence of respiratory symptoms of the child in the past week. This weekly questionnaire was identical to the survey that was used in a separate study investigating the incidence of respiratory symptoms in children who were deemed healthy by their parents [13]. This population served as a control group for this study (questionnaires used in Supplemental Table 3). If symptoms were present, the following symptoms were assessed: cough, earache, ear discharge, blocked nose, runny nose, throat ache, hoarse voice, dyspnea, headache, and fever. In addition, questions regarding the consequences of the symptoms were asked, including doctor’s visits, antibiotic treatment, and child or parental absenteeism from school or work.

Both observational studies were approved by the regional Research Ethics Board (METC Brabant, M362 and M454).

### Statistical analysis

Statistical analyses were performed using IBM SPSS statistics 26 and R v3.4.4. Descriptive analyses were conducted following two approaches. First, we evaluated all aggregated data together. In these analyses, we addressed the baseline characteristics of the cohort, as well as the total number of reported symptoms in all reported childweeks, not taking into account the longitudinal character of the data. Second, we aggregated the data per calendar week. In this approach, the year was divided into 52 weeks, where the first calendar week contains four or more days of the new year. Given the two-year duration of the study, every participant could contribute up to two times for each calendar week. However, due to missing data, participants could also contribute once or provide no data for a particular calendar week. This enabled our descriptive analyses which comprised of means, medians and percentages, and visual evaluation of data patterns.

Subsequently, we evaluated the data longitudinally by collating the weekly data for each individual participant. We determined the proportion of each symptom. For example, the number of weeks with reported cough (e.g., 4 weeks) was divided by the total number of reported weeks (e.g., 80 weeks), resulting in a proportion of 0.05. In addition, the duration of each reported symptom was determined by calculating the number of subsequent weeks for each block of weeks with the symptom present.

Given the number of symptoms assessed in this study (*k* = 10), we performed dimensionality reduction with principal component analysis (Eigenvalues > 1, maximum 25 iterations, Varimax with Kaiser normalization) on the proportions of symptoms per individual child. For this analysis, only the participants who provided data on ≥ 10 childweeks were included.

We analyzed whether subgroups of participants could be identified based on (1) the proportion of symptoms present or (2) the presence of specific combinations of symptoms. Therefore, we performed latent profile analysis for continuous data (R mclust package) to analyze the proportions of symptoms per individual child in the Down syndrome cohort. To identify subgroups of symptom combinations, we performed latent class analysis for binary data (symptom yes/no; R poLCA package) using the Akaiki information criterion (AIC) to select the best model. The latent class analysis was performed for Down syndrome, Down syndrome versus children from the general population [13], and in all data from both cohorts combined. A Pearson’s chi-squared test with Yates’ continuity correction was performed to identify significant differences in occurrence of symptoms between Down syndrome and children from the general population.

To identify patterns and potential predictors of respiratory symptoms in Down syndrome we performed a linear mixed effects regression analysis using the lme4 package in R (child as random factor), selecting the best model by using the AIC, taking main effects as well as interactions into account [13]. We analyzed four potential predictors: season (spring, summer, autumn, winter), age category at the start of the study, atopy in the family, and sex. As the dependent variable, we used the logit values of the proportions of symptoms adding 0.005 to 0-values and subtracting 0.005 from 1-values. To compare pairs of means we used the lsmeans package in R (currently emmeans).

Comparisons between various clinical characteristics of the Down syndrome and children from the general population cohorts were performed using Pearson’s correlation. Coefficients (r) between 0.1 and 0.3 were interpreted as “small,” between 0.3 and 0.5 as “medium,” and between 0.5 and 1.0 as “large” effect sizes.

Because our analyses did not account for missing data, we evaluated the significance of these missing data by (1) computing the Pearson’s correlation between the proportion of missing data and the proportion of symptoms and (2) by performing an independent *t*-test between the means of proportions of symptoms in children with a response rate of ≥ 75% (high response rate) and children with a response rate of < 75% (low response rate).

## Results

### Composition of the study cohorts

The baseline characteristics of the study cohort are displayed in Table 1. Informed consent was obtained for 131 children. However, 15 children had a 0% response rate and were excluded from the analyses. The remaining 116 children (44 girls [38%]) provided data on 9011 childweeks, reflecting an overall response rate of 75%. The Pearson’s correlation between the proportion of missing data and the proportion of symptoms was moderate (*r* = 0.366, two-tailed, *p* < 0.01), and the independent *t*-test between high and low response rate groups did not show any significant differences. Therefore, we did not take absent data into account in our analyses. The majority of the participants were less than 8 years of age (median age 5 years, interquartile range 2.2–8 years). Consistent with the age distribution, two-thirds of the participating children with Down syndrome attended a daycare facility, which could contribute to a higher burden of infection. Approximately one-third of the participants had a congenital heart defect, which is lower than expected [1]. At baseline, chronic airway problems, recurrent respiratory infections, previous ENT surgery, and frequent antibiotic use were common. Follow-up data after 1 and 2 years were non-contributory due to a high rate of non-response (23% after 1 year and 33% after 2 years; Supplemental Table 4). In the children from the general population, a total of 755 children were included (381 girls, 50.1%; median age at inclusion 7 years, interquartile range 4–11 years) with a total of 55,524 reported childweeks (70.7% response rate) [13]. The geographical distribution of the children with Down syndrome and the children from the general population was similar (Supplemental Fig. 1).

**Table 1:** Baseline characteristics of the children with Down syndrome and the children from the general population

	**Children with Down syndrome**												**Children from the general population**							
**Included children**	*n* = 131 informed consent *n* = 116 participated (*n* = 15 had a 0% response rate, these were excluded)												*n* = 761 informed consent *n* = 755 participated (*n* = 6 had a 0% response rate, these were excluded)							
**Response rate**	100% *n* = 10	75–99% *n* = 72			50–74% *n* = 9			25–49% *n* = 12				< 25% *n* = 13	100% *n* = 108	75–99% *n* = 359		50–74% *n* = 70		25–49% *n* = 86		< 25% *n* = 132
**Sex**	Male: 61 (56)					Female: 47 (44)							Male: 380 (50)				Female 381 *(50)*			
**Age**	0–2 years *n* = 37	3–6 years *n* = 34				7–10 years *n* = 22					> 10 years *n* = 14		0–2 years *n* = 84		3–6 years *n* = 289		7–10 years *n* = 190		> 10 years *n* = 192	
**Exposure to smoking**	Yes: 2 (2)	Only outside: 14 (12)				No: 89 (77)					Unknown: 11 (9)		Yes: 26 (3)		Only outside: 83 (11)		No: 646 (86)		Unknown: n/a	
**According to the parents, the child is, compared to other children**	Less often ill: 23 (20)	Just as often ill: 49 (42)				More often ill: 35 (30)					Unknown: 9 (8)		Less often ill: 23 (20)		Just as often ill: 49 (42)		More often ill: 35 (30)		Unknown: 9 (8)	
**Risk of infection when looking at weekly schedule** High: preschool, (medical) daycare, (special needs) primary education, (special needs) secondary education; Low: parents, grandparents or childminder, apprenticeship, work	High: 74 (64)		Low: 32 (28)						Unknown: 10 (8)				n/a							
**Congenital heart disease**	Yes: 37 (32) ▪ VSD: 11 (30) ▪ ASD: 7 (19) ▪ Fallot’s tetralogy: 15 (41) ▪Others: 1 (3)		No: 69 (59)						Unknown: 10 (9)				n/a							
Required surgery in the past	24 (65)		n/a						n/a				n/a							
**Hearing problems**	Yes: 39 (34)		No: 61 (53)						Unknown: 16 (13)				n/a							
**Chronic snoring**	Yes: 11 (9)		No: 87 (75)						Unknown: 18 (16)				n/a							
**Open mouth breathing**	Yes: 40 (35)		No: 55 (47)						Unknown: 21 (18)				n/a							
**Chronic airway infection**	Current problem: 36 (56)						Problem in the past: 28 (44)						n/a							
**Wheezing**	Current problem: 12 (10)						Problem in the past: 12 (10)						n/a							
**Lifetime antibiotic use**	0–5 times: 49 (42)	6–10 times: 23 (20)				> 10 times: 22 (19)					Unknown: 22 (19)		n/a							
**Prophylactic use of antibiotic**	Current use: 9 (8)			Used in the past: 8 (7)						Unknown: 22 (19)			n/a							
**Hospital admission** because of an RSV infection before the age of 2 years	23 (20)												n/a							
**ENT surgery**	Tympanic tubes: 42 (46)		Adenoidectomy: 32 (35)						Tonsillectomy: 18 (20)				n/a							

The numbers shown reflect counts (*n*), with percentages (%) in brackets

*ENT* ear-nose-throat, *VSD* ventricular septal defect, *ASD* atrial septal defect, *RSV* respiratory syncytial virus, *n/a* not applicable

**Table 2:** Results of mixed linear effects regression modeling and least-squares means in the children with Down syndrome

$_{\text{factor}}\big \backslash ^{\text{symptom}}$	Cough	Blocked nose	Runny nose	Headache	Throat ache	Dyspnea	Ear discharge	Ear ache	Hoarse voice
Main effect season			X au = wi > su		X wi > su	X sp = au = wi > su	O		O
Interaction season*age	X < 100 mo: sp = au = wi > su; ≥ 100 mo: sp = su = au < wi; au and sp: < 100 mo > ≥ 100 mo	X < 100 mo: sp = au = wi > su; ≥ 100 mo: sp = su < wi; au and sp and su: < 100 mo > ≥ 100 mo						X wi: < 100 mo > ≥ 100 mo	
Interaction season*atopy in the family				X atopy − : su = wi > sp; su and wi: atopy − > atopy +					
Main effect atopy in the family	O	O	X atopy + > atopy −		O	X atopy + > atopy −	O	O	O
Main effect age			X < 100 mo > ≥ 100 mo	O	O	X < 100 mo > ≥ 100 mo	O		O
Main effect sex	O	O	O	O	O	O	O	X girls > boys	O

*X* significant main effect (meaning the symptom frequency is significantly affected by season, age, sex, or atopy in the family)

*su* summer, *sp* spring, *au* autumn, *wi* winter, significant differences in seasonal pattern based on pairwise comparisons, *0* no interaction and no main effect, *mo* months of age at the start of the study

**Fig. 1:**
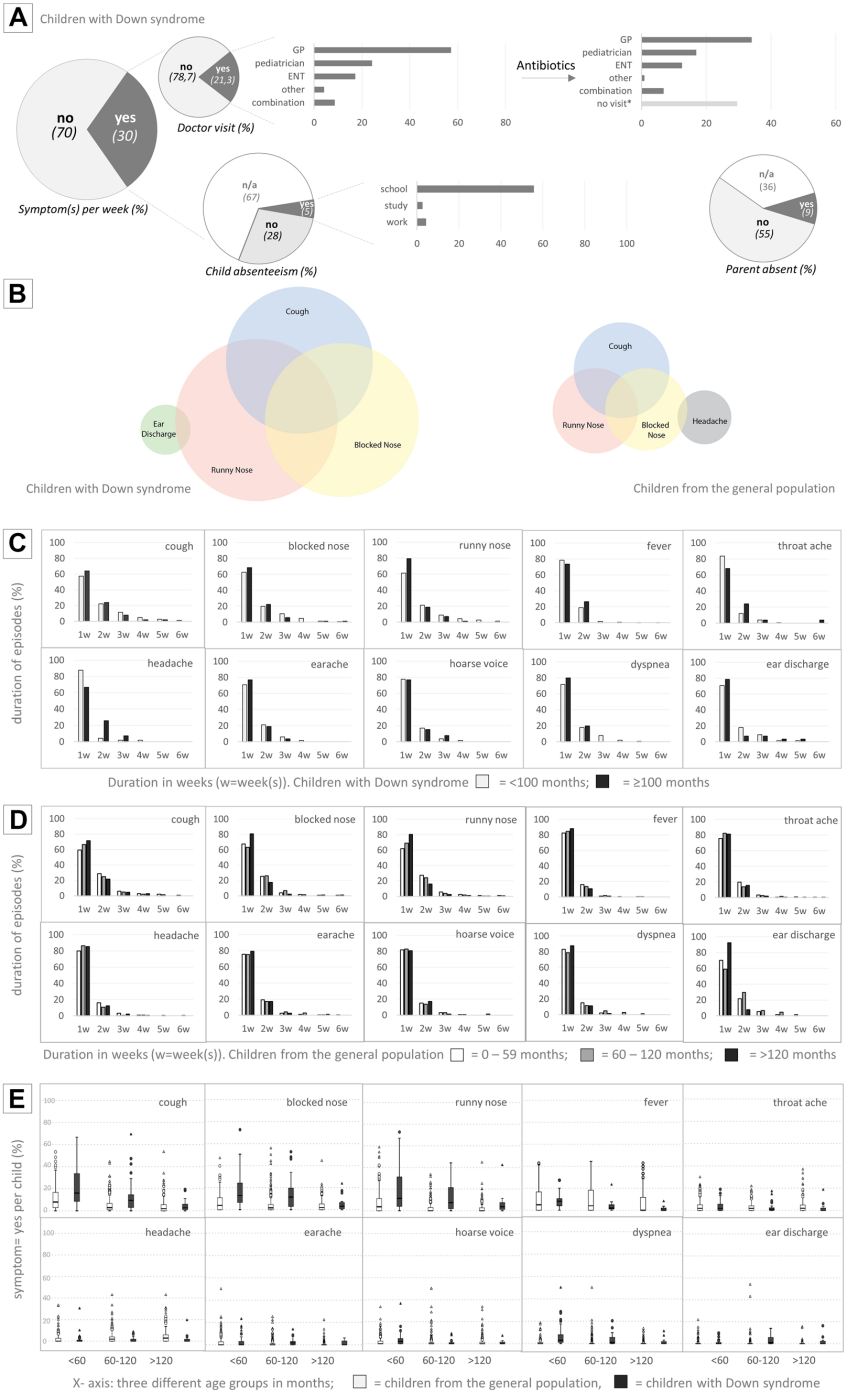
Details of symptom patterns and related actions. **A** Symptoms in the past week and related actions in children with Down syndrome. All figures are presented in percentages. n/a not applicable (answer option in the questionnaire), for example, when a child is too young or has a stay-at-home parent. **B** Venn diagrams of the most commonly occurring symptoms and their combinations of both children with Down syndrome and children from the general population. Circles are scaled to the total number of reported childweeks with symptoms. **C** Percentage of episodes with the indicated duration per symptom in children with Down syndrome. The children are divided into two age groups: 0–99 months and ≥ 100 months. **D** Percentage of episodes with the indicated duration per symptom in children from the general population. The children are divided into three age groups: 0–59 months, 60–120 months, and > 120 months. **E** Boxplots of specific symptoms in children with Down syndrome and children from the general population showing median, interquartile ranges, outliers (○), and extreme outliers (∆). *Y*-axis: percentage of symptoms “yes” per child. *X*-axis: three age groups. Children from the general population in white and children with Down syndrome in grey. To streamline the comparison, both cohorts were divided into three different age groups (0–59 months; 60–119 months, and ≥ 120 months) as used in the controls’ manuscript [13]

### Analysis of symptoms per child week

Figure 1A shows the percentage of reported respiratory symptoms for children with Down syndrome (all participants, all reported childweeks) and their consequences. In 30% of their reported childweeks, one or more symptoms were present compared to 15.2% in the children from the general population. If symptoms were present, this frequently resulted in a doctor’s visit (21.3%), antibiotics (47.8% of doctor’s visits), and absenteeism from the school of the child (55.5%). In 9% of the childweeks with symptoms, one of the parents stayed at home. However, 36% of the parents answered this question with “not applicable.” This could indicate they were a stay-at-home parent, which is frequently seen in children with disabilities [15]. In the children from the general population, only 11.8% of disease episodes resulted in a visit to a doctor, and antibiotics were prescribed less frequently (26.3%) [13].

Runny nose, blocked nose, and cough were the most reported symptoms (Supplemental Fig. 2), which often occurred together (Fig. 1B). Other symptom combinations are displayed in Supplemental Figs. 2 and 3. We divided our cohort into two age groups: < 100 months and ≥ 100 months (Supplemental Fig. 4) based on visual inspection of the data. In children from the general population, a similar cut-off point was seen at 60 months [13]. Fever was not measured in 15% of the childweeks with symptoms, and not answered in two-thirds of the reported childweeks. Therefore, we excluded fever from further analyses.

**Fig. 2:**
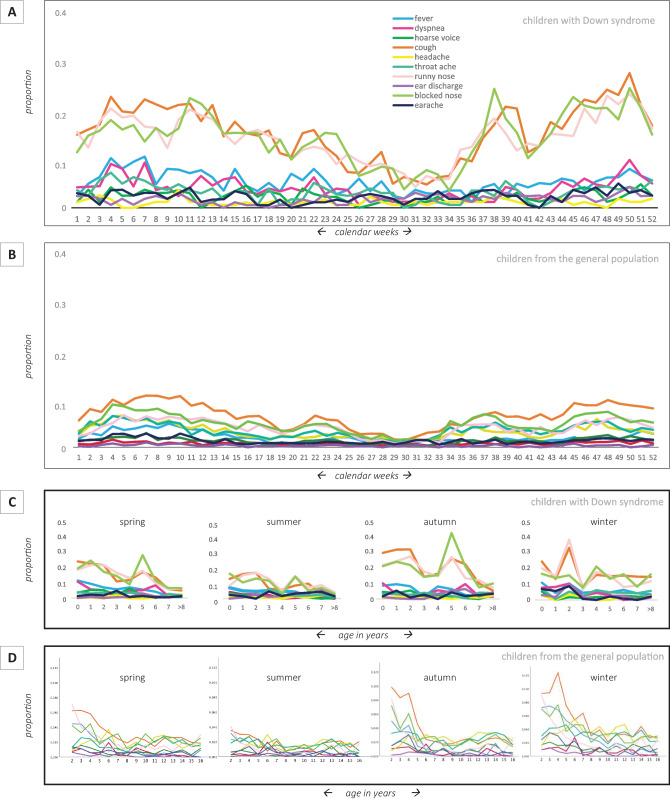
Proportion of childweeks related to season and age. **A** Proportion of childweeks in the children with Down syndrome where the symptom was “yes, present” per calendar week (1 to 52; aggregated information from 2012 to 2014; all children taken together). **B**: Proportion of childweeks in the children from the general population where the symptom was “yes, present” depicted per calendar week (1 to 52; aggregated information from years 2012 to 2015; all children taken together; (adapted from [13]). All symptoms could be reported as “yes, present” or “no, not present,” except fever which could be reported as “temperature not taken” as well. Although the various symptoms show different seasonal patterns, note the very low overall proportions of “yes, present” for all symptoms, even in winter. **C** Proportion of childweeks where the symptom was “yes, present” per season and age category in children with Down syndrome. Because of small group sizes, children aged ≥ 8 years at the start of the study were aggregated. **D** Proportion of childweeks where the symptom was “yes, present” per season and age category in children from the general population. Please note: the *y*-axes of 2C and 2D are not the same

**Fig. 3:**
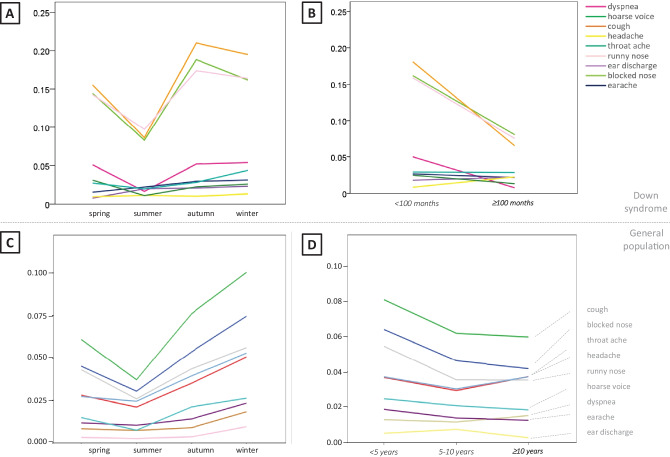
Statistical analysis of the influence of season and age. Mixed linear effects regression modeling on the logit of the proportions of childweeks with “yes, this symptom present” in children with Down syndrome in panels **A** and **B** and children from the general population in panels **C** and **D**. **A** Seasons: significant seasonal differences are seen in runny nose, blocked nose, and cough. **B** Age: significant differences are seen in runny nose, dyspnea, blocked nose, and cough. **C** Seasons: all symptoms show some seasonal differences, in some symptoms more profound than in other symptoms (cough, blocked nose, runny nose, throat ache and headache). **D** Age: significant differences are seen in cough, blocked nose and runny nose. Please note: the *y*-axes of 3**A** and **B** and 3**C** and **D** are not the same

Next, we evaluated the duration of symptoms (Fig. 1C). The majority of symptoms lasted 1 week, although up to 10% of symptom episodes lasted 3 weeks or longer, which is comparable to the children from the general population [13]. There was no noticeable difference between participants younger and older than 100 months of age at inclusion. Compared with the children from the general population, participants with Down syndrome reported more episodes of runny nose, blocked nose, and cough, especially in the younger age groups (Fig. 1D and Supplemental Fig. 5).

To explore the influence of season and age on symptom frequency, data was aggregated per calendar week. For each symptom, the proportion was calculated per calendar week (i.e., the number of participants with the symptom present divided by the total number of questionnaires for that calendar week). The proportions for Down syndrome and children from the general population are displayed in Fig. 2A, B, respectively. Higher rates of symptoms were reported for Down syndrome, but similar—limited—seasonal trends were observed. With increasing age, symptoms of runny nose, blocked nose, and cough decreased in spring, summer, and autumn, but not in winter (Fig. 2C).

### Symptom pattern analyses

Principal component analysis was performed to identify the main (combinations of) variables that determine the variance within the study population. To avoid bias, children who contributed less than 10 weeks of data were excluded from this analysis. This principal component analysis resulted in four components with loadings ≥ 600: (1) throat ache, headache, and hoarse voice; (2) cough and dyspnea; (3) earache and ear discharge; and (4) runny nose. This means, for example, that throat ache frequently occurs simultaneously with headache and hoarse voice.

As described earlier, latent class analysis and latent profile analysis were performed on binary and continuous data, respectively. No new insights on the pattern of symptoms were identified using latent class analysis. Latent profile analysis on the proportions of symptoms resulted in 2–9 best fitting clusters; however, a clear pattern could not be identified in this analysis either, both per season and year-round (data not shown).

Linear mixed effects regression analysis showed a main effect for season in dyspnea, throat ache, and runny nose, meaning that season plays a significant role in the presence of these symptoms (Table 2). Age and atopy in the family showed a main effect in runny nose and dyspnea. Only earache showed a main effect for sex. In cough, blocked nose, and earache, an interaction between season and age was found. For example, in children aged < 100 months at the start of the study, the cough was present more often in spring, autumn, and winter compared to summer. In the age group ≥ 100 months at the start of the study, the cough was present more often in winter compared to spring, autumn, and summer. For headache, there was an interaction between season and atopy in the family. Testing which differences were significant between the different seasons (spring, summer, autumn, winter) per separate symptom was performed pairwise by lsmeans (currently emmeans, see the “Methods” section and Fig. 3). Direct comparison of the proportions of symptoms (year-round) per individual child between the children with Down syndrome and the children from the general population showed a significant small effect size of having Down syndrome for cough, runny nose, blocked nose, and dyspnea (Down syndrome > general population), and headache (general population > Down syndrome). Analysis per separate season did not show significant results (data not shown).

## Discussion

Our study shows that although Down syndrome children suffer the same type of respiratory symptoms as children from the general population, they do have a higher frequency of symptoms, which supports the impression of many parents and health care professionals. In addition, this study shows that symptoms also subside at a later age (around 8 vs 5 years of age). The overall influence of season on the frequency of symptoms was limited in Down syndrome children, and comparable to children from the general population.

Recurrent respiratory symptoms have a considerable impact on overall development, health-related quality of life, and health care costs in children with Down syndrome [4, 16, 17]. Ear infections in particular have a negative impact on speech and language developmental, emotional and behavioral development, and quality of life [17, 18]. As it is known that children with Down syndrome who have a higher quality of life and fewer behavioral problems are more likely to have employment later on in life [19], these respiratory tract symptoms do not only result in short-term morbidity, they may affect long-term outcome for these individuals as well. This underlines the importance of early detection as well as appropriate treatment of respiratory symptoms.

Most viruses causing respiratory symptoms have a seasonal pattern [20]. The limited influence of season we found on these symptoms suggests they are probably not caused (or aggravated) by (viral) pathogens alone. Unfortunately, little to no evidence is available regarding the pathogens involved in respiratory tract infections in Down syndrome. The limited seasonal influence can be explained by the multiple factors that contribute to the recurrence rate and the higher risk of a severe course of respiratory infections in Down syndrome. First, the majority of children with Down syndrome have anatomical abnormalities such as midface hypoplasia, macroglossia, narrow nasopharynx and trachea, tracheal bronchus, and laryngo- or tracheomalacia [21]. Second, local physiological abnormalities such as increased mucus production and impaired ciliary function result in stasis of mucus, and generalized hypotonia contributes to insufficient mucus clearance. Third, immunological abnormalities affecting innate and adaptive immunity may contribute to an increased susceptibility to and delayed clearance of infections. Children with Down syndrome are known to have abnormalities in their B- and T-cell compartments, specific defects in B-cell memory, a lower level of IgM, IgG2, and IgG4, impaired maturation of specific antibodies, as well as poor antibody responses to vaccines [22–28]. However, until now, immunological abnormalities in Down syndrome have not consistently been correlated to respiratory disease. In addition, recent data suggest an autoinflammatory component which could increase disease severity [5, 21, 25, 29–31]. At last, it is often thought that cardiac defects contribute to or are associated with respiratory disease. However, there is insufficient evidence to support this [32, 33]. Due to the small cohort of children with cardiac defects, we were not able to compare children with and without cardiac defects in this study.

Our study has some limitations. First, our study comprises a unique, but relatively small cohort. Second, although the baseline characteristics of our cohort were similar to the children from the general population [13], it cannot be excluded that an unintended inclusion bias was present. Parents with children with Down syndrome who experience a lot of symptoms could be more motivated to take part in this research. This could affect the outcome of our cohort. However, this could also be the case for our control group and thus, in our opinion, will not have greatly impacted the results and the comparison between the two groups. Finally, the overall response rate was 75%, resulting in missing data. Because the Pearson’s correlation between the proportion of missing data and the proportion of symptoms was moderate and the independent *t*-test did not show any significant differences between means of proportions of symptoms, we concluded this had limited influence on our analyses.

In this first longitudinal prospective study, we show that children with Down syndrome suffer from respiratory symptoms more frequently than children from the general population and that these symptoms subside more slowly with age compared to children from the general population. The overall characteristics (type, duration, and pattern) of these symptoms are comparable between groups. Given the complex nature of respiratory symptoms in Down syndrome, treatment should be targeted and individualized to prevent unfavorable short- and long-term adverse outcomes, while avoiding unnecessary treatments. Unfortunately, there is a lack of interventional studies on the optimal management of these symptoms, which puts these children at risk for over- as well as undertreatment. Further research should focus on the evaluation and development of diagnostic tools to identify the main contributing factors to respiratory disease in these children to optimize management strategies.

## Supplementary Information

Below is the link to the electronic supplementary material.

Supplementary file1 (PDF 176 KB)

Supplementary file2 (PDF 176 KB)

Supplementary file3 (PDF 166 KB)

Supplementary file4 (PDF 253 KB)

Supplementary file5 (PDF 237 KB)

Supplementary file6 (PDF 185 KB)

Supplementary file7 (PDF 369 KB)

Supplementary file8 (PDF 223 KB)

Supplementary file9 (PDF 208 KB)

## Data Availability

After publication, the data will be available to researchers upon reasonable request. Depositing the data in a public repository was not part of the informed consent signed by the parents.

## Abbreviations

AIC: Akaike information criterion
ENT: Ear-nose-throat
