# Supplementary material for: Increased rate of respiratory symptoms in children with Down syndrome: a 2-year web-based parent-reported prospective study
Source: Eur J Pediatr. 2022 Oct 3;181(12):4079–89. doi: 10.1007/s00431-022-04634-1 (PMC9649482; doi:10.1007/s00431-022-04634-1)
Supplement: Supplementary file 1 — Supplementary file1 (PDF 176 KB) [file 431_2022_4634_MOESM1_ESM.pdf]

Supplemental Table 1: STROBE Statement—Checklist KiDS study

| | Item No | Recommendation | Page No |
|---|---|---|---|
| **Title and abstract** | 1 | (*a*) Indicate the study's design with a commonly used term in the title or the abstract<br>(*b*) Provide in the abstract an informative and balanced summary of what was done and what was found | 1, 2 |
| **Introduction** | | | |
| Background/rationale | 2 | Explain the scientific background and rationale for the investigation being reported | 4 |
| Objectives | 3 | State specific objectives, including any prespecified hypotheses | 4 |
| **Methods** | | | |
| Study design | 4 | Present key elements of study design early in the paper | 5 |
| Setting | 5 | Describe the setting, locations, and relevant dates, including periods of recruitment, exposure, follow-up, and data collection | 5 |
| Participants | 6 | (*a*) Give the eligibility criteria, and the sources and methods of selection of participants. Describe methods of follow-up<br>(*b*) For matched studies, give matching criteria and number of exposed and unexposed | 5 |
| Variables | 7 | Clearly define all outcomes, exposures, predictors, potential confounders, and effect modifiers. Give diagnostic criteria, if applicable | 5* |
| Data sources/ measurement | 8 | For each variable of interest, give sources of data and details of methods of assessment (measurement). Describe comparability of assessment methods if there is more than one group | 5 |
| Bias | 9 | Describe any efforts to address potential sources of bias | ** |
| Study size | 10 | Explain how the study size was arrived at | *** |
| Quantitative variables | 11 | Explain how quantitative variables were handled in the analyses. If applicable, describe which groupings were chosen and why | 6-8 |
| Statistical methods | 12 | (*a*) Describe all statistical methods, including those used to control for confounding<br>(*b*) Describe any methods used to examine subgroups and interactions<br>(*c*) Explain how missing data were addressed<br>(*d*) If applicable, explain how loss to follow-up was addressed<br>(*e*) Describe any sensitivity analyses | 6-8 |
| **Results** | | | |
| Participants | 13 | (a) Report numbers of individuals at each stage of study—eg numbers potentially eligible, examined for eligibility, confirmed eligible, included in the study, completing follow-up, and analysed<br>(b) Give reasons for non-participation at each stage<br>(c) Consider use of a flow diagram | 8 |
| Descriptive data | 14 | (a) Give characteristics of study participants (eg demographic, clinical, social) and information on exposures and potential confounders<br>(b) Indicate number of participants with missing data for each variable of interest<br>(c) Summarise follow-up time (eg, average and total amount) | 8 |
| Outcome data | 15 | Report numbers of outcome events or summary measures over time | 8 |

| | | | |
|---|---|---|---|
| Main results | 16 | (*a*) Give unadjusted estimates and, if applicable, confounder-adjusted estimates and their precision (eg, 95% confidence interval). Make clear which confounders were adjusted for and why they were included<br>(*b*) Report category boundaries when continuous variables were categorized<br>(*c*) If relevant, consider translating estimates of relative risk into absolute risk for a meaningful time period | 8 |
| Other analyses | 17 | Report other analyses done—eg analyses of subgroups and interactions, and sensitivity analyses | 9-11 |
| **Discussion** | | | |
| Key results | 18 | Summarise key results with reference to study objectives | 11,12 |
| Limitations | 19 | Discuss limitations of the study, taking into account sources of potential bias or imprecision. Discuss both direction and magnitude of any potential bias | 13 |
| Interpretation | 20 | Give a cautious overall interpretation of results considering objectives, limitations, multiplicity of analyses, results from similar studies, and other relevant evidence | 13 |
| Generalisability | 21 | Discuss the generalisability (external validity) of the study results | **** |
| **Other information** | | | |
| Funding | 22 | Give the source of funding and the role of the funders for the present study and, if applicable, for the original study on which the present article is based | 3 |

* In our cohort of children with Down syndrome, several confounders were analyses (congenital heart disease, smoking etc.). This is not explicitly explained in the article (see supplemental document 1)
** This is addressed in the previous article "Epidemiology of respiratory symptoms in children with Down syndrome: a nationwide prospective web-based parent-reported study" by Verstegen et al in the BMC Pediatrics 2014, 14:103. Page 2.
*** This is addressed in the previous article "Epidemiology of respiratory symptoms in children with Down syndrome: a nationwide prospective web-based parent-reported study" by Verstegen et al in the BMC Pediatrics 2014. Page 2.
**** not applicable

*Increased rate of respiratory symptoms in children with Down syndrome: a 2-year web-based parent-reported prospective study, European Journal of Pediatrics,* Esther de Vries, MD PhD.*Tranzo, Tilburg School of Social and Behavioral Sciences, Tilburg University, Tilburg, the Netherlands; Jeroen Bosch Academy Research, Jeroen Bosch Hospital, 's-Hertogenbosch, the Netherlands.* **Correspondence:** Esther de Vries, MD PhD, Tranzo, TSB, Tilburg University, PO Box 90153 (RP219), 5000LE Tilburg, the Netherlands, e.devries@tilburguniversity.edu, Telephone number: +31 (0)13 466 2969.
