# Supplementary material for: Increased rate of respiratory symptoms in children with Down syndrome: a 2-year web-based parent-reported prospective study
Source: Eur J Pediatr. 2022 Oct 3;181(12):4079–89. doi: 10.1007/s00431-022-04634-1 (PMC9649482; doi:10.1007/s00431-022-04634-1)
Supplement: Supplementary file 2 — Supplementary file2 (PDF 176 KB) [file 431_2022_4634_MOESM2_ESM.pdf]

Supplemental Table 2: Annual questionnaire regarding background, daily activities and medical history of participating child with Down syndrome

**General questions**			
Child with Down syndrome	Date of birth<br>Sex		
Father/mother	Date of birth		
History of allergy, asthma and/or eczema	Yes	No	
Siblings	Number of older siblings		
History of allergy, asthma and/or eczema	Yes	No	
Does anyone smoke (almost) daily within the house?	Yes	No, only outside	No
**Daily activities**			
Divide the 14 half-days present in each week between the following activities	Home	Grandparents/family/host family	
	Child day care	Special needs day care	
	Playgroup (age 2-4y)	Pre-school kindergarten (age 4-5y)	
	Primary school (age 6-12y)	Special primary school	
	Secondary school	Special secondary school	
	Work placement<br>Other	Working	
If attending regular education, what grade is your child in?			
**Past Medical History**			
Compared to other children with the same age, the frequency of being ill is:	Lower Equal	Higher	
Does your child have a history of any of the following illnesses, complaints or medication usage?			
Congenital heart disease	Yes No		
If yes, please specify	VSD ASD AVSD	tetralogy of Fallot other	unknown
If yes, was surgery performed	Yes No		
Hypothyroidism	Yes No		
If yes, diagnosed at what age			
Diabetes mellitus	Yes No		
If yes, diagnosed at what age			
Congenital malformations of the gastro-intestinal tract	Yes No		

If yes, please specify	esophageal atresia		duodenal atresia	imperforate anus
	other		unknown	
Celiac disease	Yes	No		
If yes, diagnosed at what age				
Impaired hearing	Yes	No		
If yes, diagnosed at what age				
Chronic snoring	Yes	No		
If yes, diagnosed at what age				
Breathing with open mouth	Yes	No		
If yes, present since what age				
Frequently suffering from serious colds	Yes	No, but did in the past	No	
If complaints used to be present, until what age?				
Wheezing	Yes	No, but did in the past	No	
If complaints used to be present, until what age?				
Eye disorders	Yes	No		
If yes, please specify	cataract		glaucoma	strabismus
	amblyopia		wears glasses	other
	unknown			
Leukemia	Yes	No		
If yes, diagnosed at what age?				
Antibiotic use for respiratory tract/ENT infections in the past year	0-5 times	6-10 times	More than 10 times	
Hospital admission for RSV infection <2 years	Yes	No		
ENT surgery	Yes	No		
If yes, please specify	Tympanic tubes		adenoidectomy	tonsillectomy
Daily antibiotic prophylaxis	Yes	No, but did in the past	No	
Inhaled corticoid for coughing, mucus and/or wheezing	Yes	No, but did in the past	No	

ENT, ear-nose-throat

Supplemental Table 2: Weekly questionnaire regarding medical symptoms in the past week

**Did your child have any symptoms in the past week?**	**No**	**Yes**	
If yes,*			
Did you visit a doctor with your child?	No		Yes, general practitioner
	Yes, pediatrician		Yes, ENT specialist
	Yes, other doctor		
Did your child receive antibiotic treatment?	No	Yes	
Which symptoms were present?	earache	ear discharge	sore throat
	blocked nose<br>hoarse voice	runny nose<br>coughing/mucus	headache
Was the temperature higher than 38.5°C (fever)?	No	Yes	Did not take a temperature
Did your child stay at home from school?	No	Yes	Not applicable
Did your child stay at home from work placement?	No	Yes	Not applicable
Did your child stay at home from work?	No	Yes	Not applicable
Did you or your partner stay at home from work?	No	Yes	Not applicable

* The additional questions were only visible if the previous question was answered with yes. ENT, ear-nose-throat

Increased rate of respiratory symptoms in children with Down syndrome: a 2-year web-based parent-reported prospective study, European Journal of Pediatrics, Esther de Vries, MD PhD.*Tranzo, Tilburg School of Social and Behavioral Sciences, Tilburg University, Tilburg, the Netherlands; Jeroen Bosch Academy Research, Jeroen Bosch Hospital, ‘s-Hertogenbosch, the Netherlands.* **Correspondence:** Esther de Vries, MD PhD, Tranzo, TSB, Tilburg University, PO Box 90153 (RP219), 5000LE Tilburg, the Netherlands, e.devries@tilburguniversity.edu, Telephone number: +31 (0)13 466 2969.
