# Supplementary material for: Increased rate of respiratory symptoms in children with Down syndrome: a 2-year web-based parent-reported prospective study
Source: Eur J Pediatr. 2022 Oct 3;181(12):4079–89. doi: 10.1007/s00431-022-04634-1 (PMC9649482; doi:10.1007/s00431-022-04634-1)
Supplement: Supplementary file 3 — Supplementary file3 (PDF 166 KB) [file 431_2022_4634_MOESM3_ESM.pdf]

Supplementary Table 3. Online questionnaire at start of the inclusion of the children from the general population (Kind-en-Ziek)*

**General questions**	
Date of birth	dd/mm/yyyy
Gender	male / female
First 2 digits of postal code	*(Dutch postal codes contain 4 digits followed by 2 letters; the first 2 digits define the geographical area, but give no clue to the exact address)*
Ethnicity	Original Dutch / Surinam / Netherlands Antilles / Indonesian / Moluccan / Moroccan / Turkish / Other Asian / Other African / Latino / Mixed / I prefer not to answer this question
Allergy, asthma or eczema in the family?	no / yes / don’t know
Frequent infections in the family?	no / yes / don’t know
Smoking in the home?	no / no, only outside / yes, but only by visitors / yes, by family members

Supplemental Table 3: Weekly Questionairre (weeks 1-104)

**Did your child have any complaints last week?**	**no / yes**
If yes: the questions below followed	
Did you visit a doctor (please tick applicable items)?	no / yes, general practitioner / yes, paediatrician / yes, ENT-specialist / yes, other
Did your child get antibiotics?	no / yes
What were the complaints (please tick applicable items)?	earache / ear discharge / throat ache / blocked nose / runny nose / headache / hoarse voice / cough / dyspnoea
Was there a fever ≥ 38.5°C?	no / yes / didn’t take the temperature

Did the child miss school?	no / yes / not applicable
Did the child miss study work placement (*'stage' in Dutch*)?	no / yes / not applicable
Did the child miss work?	no / yes / not applicable
Did the parents miss their work?	no / yes / not applicable

*Original questionnaires *in Dutch*. Population cohort is explained in the methods section of the manuscript. ENT = ear-nose-throat.

Increased rate of respiratory symptoms in children with Down syndrome: a 2-year web-based parent-reported prospective study, European Journal of Pediatrics, Esther de Vries, MD PhD.*Tranzo, Tilburg School of Social and Behavioral Sciences, Tilburg University, Tilburg, the Netherlands; Jeroen Bosch Academy Research, Jeroen Bosch Hospital, 's-Hertogenbosch, the Netherlands.* **Correspondence:** Esther de Vries, MD PhD, Tranzo, TSB, Tilburg University, PO Box 90153 (RP219), 5000LE Tilburg, the Netherlands, e.devries@tilburguniversity.edu, Telephone number: +31 (0)13 466 2969.
