# Supplementary material for: Increased rate of respiratory symptoms in children with Down syndrome: a 2-year web-based parent-reported prospective study
Source: Eur J Pediatr. 2022 Oct 3;181(12):4079–89. doi: 10.1007/s00431-022-04634-1 (PMC9649482; doi:10.1007/s00431-022-04634-1)
Supplement: Supplementary file 4 — Supplementary file4 (PDF 253 KB) [file 431_2022_4634_MOESM4_ESM.pdf]

Supplementary Table 1. Baseline and follow-up characteristics of the cohort.

	N *(%)* at the start[1]	N *(%)* after 1 year[1]	N *(%)* at the end[1]
**Sex**			
- Male	61 *(56)*		
- Female	47 *(44)*		
**Exposure to smoking**			
- Yes	2 *(2)*	0 *(0)*	1 *(1)*
- Only outside the house	14 *(12)*	17 *(15)*	11 *(9)*
- No	89 *(77)*	68 *(58)*	65 *(56)*
- Unknown	11 *(9)*	31 *(27)*	39 *(34)*
**Risk of infection when looking at weekly schedule**			
- High (preschool, [medical] daycare, [special needs] primary education, [special needs] secondary education)	74 *(64)*	59 *(51)*	58 *(50)*
- Low (parents, grandparents or caregiver, apprenticeship, work)	32 *(28)*	20 *(18)*	13 *(11)*
- Unknown	10 *(8)*	36 *(31)*	45 *(39)*
**According to the parents, the child is:**			
- Less often ill compared to other children	23 *(20)*	21 *(18)*	20 *(17)*
- Just as often ill as other children	49 *(42)*	42 *(36)*	35 *(30)*
- More often ill compared to other children	35 *(30)*	24 *(21)*	21 *(18)*
- Unknown	9 *(8)*	29 *(25)*	40 *(35)*
**Congenital heart disease**			
- Yes	37 *(32)*		
○ VSD	11 *(30)*		
○ ASD	7 *(19)*		
○ AVSD	15 *(41)*		
○ Fallot's tetralogy	1 *(3)*		

○ Others<br>○ *Required surgery in the past (start) or past year (at 1&2 years)*<br>- No<br>- Unknown	3 *(8)*<br>24 *(65)*<br><br>69 *(59)*<br>10 *(9)*	3 *(3)*	2 *(3)*
**Hypothyroidism**<br>- Yes<br>- Unknown	 9 *(8)*<br>11 *(9)*		
**Diabetes mellitus**	0 *(0)*		
**Gastro-intestinal diseases**<br>Yes<br>- Esophageal atresia<br>- Duodenal atresia<br>- Anal atresia<br>- Other<br>No<br>Unknown	 6 *(5)*<br>1 *(17)*<br>1 *(17)*<br>1 *(17)*<br>3 *(50)*<br>95 *(82)*<br>15 *(13)*		
**Celiac disease**<br>Yes<br>Unknown	 3 *(3)*<br>16 *(14)*		
**Hearing problems**<br>Yes<br>- Yes, diagnosed since past year<br>No<br>Unknown	 39 *(34)*<br><br>61 *(53)*<br>16 *(13)*	10 *(9)*	7 *(6)*
**Chronic snoring**<br>Yes<br>- Yes, diagnosed since past year<br>No<br>Unknown	 11 *(9)*<br><br>87 *(75)*<br>18 *(16)*	1 *(1)*	3 *(3)*

**Open mouth breathing**			
Yes	40 *(35)*		
- Yes, diagnosed since past year		4 *(3)*	1 *(1)*
No	55 *(47)*		
Unknown	21 *(18)*		
**Chronic airway infections**			
- Current problem	36 *(56)*	7 *(6)*	4 *(3)*
- Problem in the past	28 *(44)*	28 *(24)*	29 *(25)*
**Wheezing**			
- Current problem	12 *(10)*	2 *(2)*	1 *(1)*
- Problem in the past	12 *(10)*	10 *(9)*	7 *(6)*
**Eye problems**			
Yes	44 *(37)*		
- Cataract	1 *(2)*		
- Glaucoma	0 *(0)*		
- Strabismus	10 *(23)*		
- Amblyopia	7 *(16)*		
- Corrective glasses	33 *(75)*		
- Other	7 *(16)*		
No	51 *(43)*		
Unknown	21 *(18)*		
**Leukemia**			
Yes	1 *(1)*		
- Yes, diagnosed since past year		0	0
No	93 *(80)*		
Unknown	22 *(19)*		
**Antibiotic use**			
- 0-5 times	49 *(42)*	79 *(68)* [2]	69 *(59)* [2]
- 6-10 times	23 *(20)*	4 *(3)* [2]	3 *(3)* [2]
- More than 10 times	22 *(19)*	3 *(3)* [2]	0 *(0)* [2]
- Unknown	22 *(19)*	30 *(26)*	43 *(37)*
- Prophylactic use			
○ Current use	9 *(8)*	1 *(1)*[2]	0 *(0)* [2]

○ Used in the past<br>○ Unknown	8 *(7)*<br>22 *(19)*	7 *(6)*[3]<br>31 *(27)*	7 *(6)*[3]<br>44 *(38)*
**Hospital admission** because of an RSV infection before the age of 2 years	23 *(20)*	7 *(6)*[4]	3 *(3)*[4]
**ENT surgery**<br>- Tympanic tubes<br>- Adenoidectomy<br>- Tonsillectomy	 42 *(46)*<br>32 *(35)*<br>18 *(20)*	 22 *(23)*[4]<br>10 *(10)*[4]<br>4 *(4)*[4]	 14 *(50)*[4]<br>7 *(25)*[4]<br>7 *(25)*[4]
**Inhaled corticosteroids**<br>- Current use<br>- Used in the past	 7 *(6)*<br>19 *(16)*	 5 *(4)*[4]<br>4 *(3)*[4]	 1 *(1)*[4]<br>5 *(4)*[4]

Supplementary Table 1. Baseline and follow-up characteristics of the cohort.

The amounts presented are numbers, with percentages in brackets.

[1]Parents answered an extended questionnaire when they started to participate in the study, one year after they started to participate, and at the end of the study period (two years after they had started).

[2]Question answered based on the situation in the year before the questionnaire.

[3]Some of whom are still using prophylactic antibiotics.

[4]Only containing data from the preceding year.

Increased rate of respiratory symptoms in children with Down syndrome: a 2-year web-based parent-reported prospective study, European Journal of Pediatrics, Esther de Vries, MD PhD.*Tranzo, Tilburg School of Social and Behavioral Sciences, Tilburg University, Tilburg, the Netherlands; Jeroen Bosch Academy Research, Jeroen Bosch Hospital, 's-Hertogenbosch, the Netherlands.* **Correspondence:** Esther de Vries, MD PhD, Tranzo, TSB, Tilburg University, PO Box 90153 (RP219), 5000LE Tilburg, the Netherlands, e.devries@tilburguniversity.edu, Telephone number: +31 (0)13 466 2969.
