# Supplementary material for: Increased rate of respiratory symptoms in children with Down syndrome: a 2-year web-based parent-reported prospective study
Source: Eur J Pediatr. 2022 Oct 3;181(12):4079–89. doi: 10.1007/s00431-022-04634-1 (PMC9649482; doi:10.1007/s00431-022-04634-1)
Supplement: Supplementary file 6 — Supplementary file6 (PDF 185 KB) [file 431_2022_4634_MOESM6_ESM.pdf]

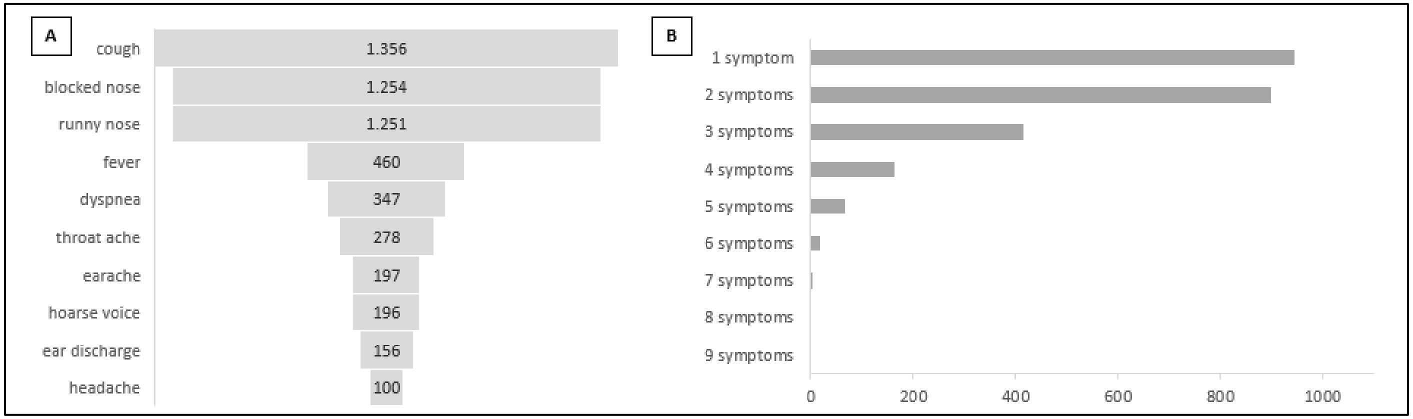


C

| | earache | ear discharge | blocked nose | runny nose | throat ache | hoarse voice | cough | dyspnea | headache |
|---|---|---|---|---|---|---|---|---|---|
| earache | | 37 | 86 | 80 | 53 | 20 | 88 | 25 | 12 |
| ear discharge | 37 | | 42 | 39 | 9 | 6 | 34 | 8 | 5 |
| blocked nose | 86 | 42 | | 485 | 146 | 120 | 653 | 166 | 55 |
| runny nose | 80 | 39 | 485 | | 121 | 92 | 640 | 136 | 34 |
| throat ache | 53 | 9 | 146 | 121 | | 73 | 195 | 73 | 41 |
| hoarse voice | 20 | 6 | 120 | 92 | 73 | | 150 | 62 | 15 |
| cough | 88 | 34 | 653 | 640 | 195 | 150 | | 287 | 48 |
| dyspnea | 25 | 8 | 166 | 136 | 73 | 62 | 287 | | 8 |
| headache | 12 | 5 | 55 | 34 | 41 | 15 | 48 | 8 | |

**Supplemental Figure 2: a: number of childweeks with (combinations of) symptoms in children with Down syndrome. b: number of symptoms per childweek (with symptoms) in children with down syndrome**. In a+b: All childweeks taken together. **c: combination of symptoms which often occur simultaneously in children with down syndrome.**

*Increased rate of respiratory symptoms in children with Down syndrome: a 2-year web-based parent-reported prospective study, European Journal of Pediatrics,* Esther de Vries, MD PhD.*Tranzo, Tilburg School of Social and Behavioral Sciences, Tilburg University, Tilburg, the Netherlands; Jeroen Bosch Academy Research, Jeroen Bosch Hospital, 's-Hertogenbosch, the Netherlands.* **Correspondence:** Esther de Vries, MD PhD, Tranzo, TSB, Tilburg University, PO Box 90153 (RP219), 5000LE Tilburg, the Netherlands, e.devries@tilburguniversity.edu, Telephone number: +31 (0)13 466 2969.
