# Supplementary material for: Increased rate of respiratory symptoms in children with Down syndrome: a 2-year web-based parent-reported prospective study
Source: Eur J Pediatr. 2022 Oct 3;181(12):4079–89. doi: 10.1007/s00431-022-04634-1 (PMC9649482; doi:10.1007/s00431-022-04634-1)
Supplement: Supplementary file 7 — Supplementary file7 (PDF 369 KB) [file 431_2022_4634_MOESM7_ESM.pdf]

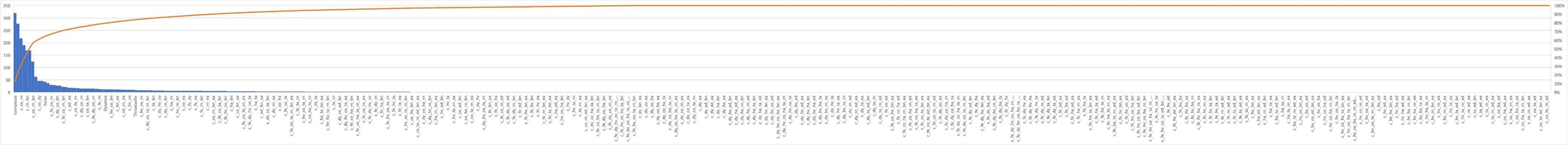

Supplemental Figure 3: Different combinations of symptoms in children with Down syndrome. This figure shows the different combinations of symptoms present. Rn = runny nose; co = cough; bn = blocked nose; dy = dyspnea; fe = fever; ed = ear discharge; ea = earache; ta = throat ache; hv = hoarse voice; ha = headache; Figure 1B shows a summary of the most common symptoms occurring together.

Increased rate of respiratory symptoms in children with Down syndrome: a 2-year web-based parent-reported prospective study, European Journal of Pediatrics, Esther de Vries, MD PhD.*Tranzo, Tilburg School of Social and Behavioral Sciences, Tilburg University, Tilburg, the Netherlands; Jeroen Bosch Academy Research, Jeroen Bosch Hospital, 's-Hertogenbosch, the Netherlands.* **Correspondence:** Esther de Vries, MD PhD, Tranzo, TSB, Tilburg University, PO Box 90153 (RP219), 5000LE Tilburg, the Netherlands, e.devries@tilburguniversity.edu, Telephone number: +31 (0)13 466 2969.
