# Supplementary material for: Increased rate of respiratory symptoms in children with Down syndrome: a 2-year web-based parent-reported prospective study
Source: Eur J Pediatr. 2022 Oct 3;181(12):4079–89. doi: 10.1007/s00431-022-04634-1 (PMC9649482; doi:10.1007/s00431-022-04634-1)
Supplement: Supplementary file 8 — Supplementary file8 (PDF 223 KB) [file 431_2022_4634_MOESM8_ESM.pdf]

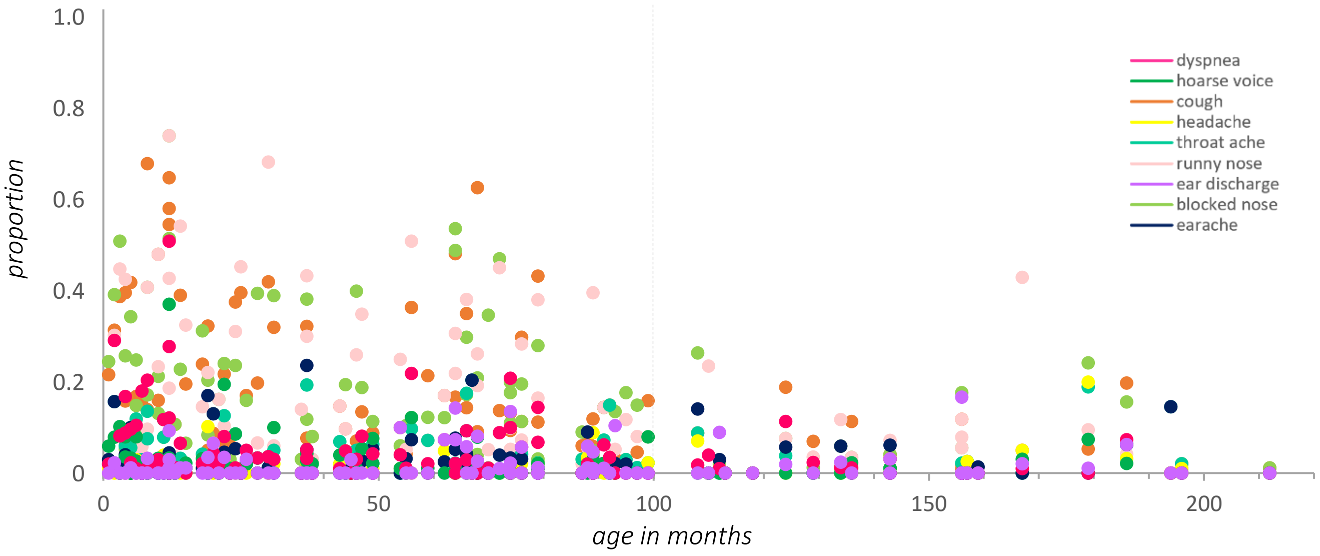

Supplemental Figure 4: Distribution of symptoms with age in children with Down syndrome. This figure shows the higher proportions predominate in the younger age group. The dotted line represents the cut off at 100 months of age. Each symptom type is depicted by a different color. Each dot represents the combination of age in months at inclusion on the x-axis, and proportion of symptoms 'yes' per individual child per separate symptom type on the y-axis.

Increased rate of respiratory symptoms in children with Down syndrome: a 2-year web-based parent-reported prospective study, European Journal of Pediatrics, Esther de Vries, MD PhD.*Tranzo, Tilburg School of Social and Behavioral Sciences, Tilburg University, Tilburg, the Netherlands; Jeroen Bosch Academy Research, Jeroen Bosch Hospital, 's-Hertogenbosch, the Netherlands.* **Correspondence:** Esther de Vries, MD PhD, Tranzo, TSB, Tilburg University, PO Box 90153 (RP219), 5000LE Tilburg, the Netherlands, e.devries@tilburguniversity.edu, Telephone number: +31 (0)13 466 2969.
